# Soy Isoflavones Induce Feminization of Japanese Eel (*Anguilla japonica*)

**DOI:** 10.3390/ijms24010396

**Published:** 2022-12-26

**Authors:** Hiroyuki Inaba, Yuzo Iwata, Takashi Suzuki, Moemi Horiuchi, Ryohei Surugaya, Shigeho Ijiri, Ai Uchiyama, Ryoko Takano, Seiji Hara, Takashi Yazawa, Takeshi Kitano

**Affiliations:** 1 Freshwater Resource Research Center, Aichi Fisheries Research Institute, Isshiki, Nishio 444-0425, Aichi, Japan; 2 Department of Biological Sciences, Graduate School of Science and Technology, Kumamoto University, Kumamoto 860-8555, Kumamoto, Japan; 3 Fisheries Administration Division, Bureau of Agriculture and Fisheries, Aichi Prefectural Governmental Office, 3-1-2 Sannomaru, Nakaku, Nagoya 460-8501, Aichi, Japan; 4 Nishimikawa Agriculture, Forestry, and Fisheries Office of Aichi Prefectural Government, Myoudaijihonmachi, Okazaki 444-0860, Aichi, Japan; 5 Marine Resources Research Center, Aichi Fisheries Research Institute, Toyohama, Minamichita 470-3412, Aichi, Japan; 6 Graduate School of Fisheries Sciences, Hokkaido University, Hakodate 041-8611, Hokkaido, Japan; 7 Advanced Technology Development Center, Kyoritsu Seiyaku Corporation, 2-9-22 Takamihara, Tsukuba 300-1252, Ibaraki, Japan; 8 Fukui Prefectural Fish Farming Center, 50-1 Katsumi, Obama 917-0166, Fukui, Japan; 9 Department of Biochemistry, Asahikawa Medical University, Asahikawa 078-8510, Hokkaido, Japan

**Keywords:** feminization, sex differentiation, Japanese eel, phytoestrogen, soy isoflavone, genistein

## Abstract

Under aquaculture conditions, Japanese eels (*Anguilla japonica*) produce a high percentage of males. However, females gain higher body weight and have better commercial value than males, and, therefore, a high female ratio is required in eel aquaculture. In this study, we examined the effects of isoflavones, genistein, and daidzein on sex differentiation and sex-specific genes of eels. To investigate the effects of these phytoestrogens on the gonadal sex, we explored the feminizing effects of soy isoflavones, genistein, and daidzein in a dose-dependent manner. The results showed that genistein induced feminization more efficiently than daidzein. To identify the molecular mechanisms of sex-specific genes, we performed a comprehensive expression analysis by quantitative real-time PCR and RNA sequencing. Phenotypic males and females were produced by feeding elvers a normal diet or an estradiol-17β- or genistein-treated diet for 45 days. The results showed that female-specific genes were up-regulated and male-specific genes were down-regulated in the gonads, suggesting that genistein induces feminization by altering the molecular pathways responsible for eel sex differentiation.

## 1. Introduction

In vertebrates, sex is determined by genotype in many species, but in poikilotherms, such as reptiles, amphibians, and fish, sex determination is greatly influenced by ambient temperature [1]. In addition, environmental factors such as pH, density, and social factors influence sex determination in some teleosts [2]. However, the molecular mechanisms of environmental sex determination in these species are largely unknown.

Japanese eel (*Anguilla japonica*) is an important species in the aquaculture industry in Japan. The seed used in aquaculture is taken from wild glass eels captured from the mouth of a river. Since the 1970s, the glass eel catch has been dramatically declining. Under cultural conditions, the sex ratio of this species becomes more biased towards males than it is under field conditions [3,4]. However, the mechanism underlying this male bias in cultured eels is unknown. A previous study of Japanese eels found that females grow larger than males [4]. Because females gain higher body weight, they have better commercial value than males, and, therefore, a high female ratio is required in eel aquaculture.

It was hypothesized that sex determination in fish is controlled by the ratio of androgen to estrogen levels at a critical period of sex determination and that steroid intervention at this period changes this balance [5]. Sex control using steroid hormones has been investigated in various fish species [2,6,7,8]. Fish are mostly divergent in sex determination and sex differentiation regulated by a cascade of sex-related genes. Estrogen treatments have been found to induce female-related genes and inhibit male-related genes in fish [9]. For example, the expression level of forkhead box L2 (*foxl2*), a key gene for ovary development, was up-regulated after treatment with estradiol in rare minnow (*Gobiocypris rarus*) [10]. In zebrafish (*Danio rerio*), exposure to 17α-ethinylestradiol during early life suppressed the expression of male-related genes and led to cessation/retardation in male gonadal sex development [11]. In eels, feminization was induced by oral treatment of elvers with estrogen [3,12,13]. Estrogen treatment was shown to increase the female-related genes *foxl2a* and the cytochrome P450 aromatase *cyp19a1* and suppress the male-related genes anti-Müllerian hormone (*amh*) and gonadal soma-derived factor (*gsdf*) in Japanese eels [14]. Aromatase converts androgens to estrogens and is thought to play an essential role in ovarian development in vertebrates [8,15]. The male-related genes *amh* and *gsdf* are members of the transforming growth factor-β (TGF-β) family and are related to testes development in fish [16,17]. Thus, we assume that feminization by estrogen is caused by the induction of female-related genes and suppression of male-related genes.

Phytoestrogens are plant-derived compounds that have estrogenic activity [18]. Soybean, which is widely used as a major protein source for farmed fish, contains phytoestrogens, including mainly genistein and daidzein [19]. Phytoestrogens contain a phenolic ring, which is a prerequisite for binding to estrogen receptors. Genistein can bind to mammalian and fish estrogen receptors and has both agonistic and antagonistic effects that affect numerous reproductive processes [20,21,22,23,24]. In Japanese medaka (*Oryzias latipes*), genistein exposure induced feminization of genetic males [25], whereas, in channel catfish (*Ictalurus punctatus*), genistein treatment during the period of sex differentiation led to a higher proportion of male and intersex fish [24]. In Russian sturgeon (*Acipenser gueldenstaedtii*), the dietary phytoestrogens genistein, daidzein, and coumestrol were shown to modulate sex-related genes, leading to endocrine effects [26]. In European eels (*Anguilla anguilla*) fed 2 mg/kg genistein for 100 days, the proportion of females was 55%, but at 20 mg/kg, the proportion of females was only 15% [27]. Together, these results suggested that phytoestrogens can be used as alternatives to gonadal steroids for sex control in eels, but suitable concentrations and treatment periods remain to be determined. Moreover, the effect of phytoestrogens on the expression of sex-related genes is poorly understood in fish.

In this study, we investigated the effects of isoflavones on gonadal sex differentiation in Japanese eels by examining the expression patterns of sex-related genes in the gonads of isoflavone-treated Japanese eels. First, we investigated the feminization effects of soy isoflavones and the expression patterns of sex-specific genes. Next, we clarified the concentration-dependent feminization effects of genistein. Then, we investigated the feminization mechanism of estradiol-17β (E2) and genistein.

## 2. Results

### 2.1. Sex Ratio and Gene Expression Patterns in Control and Soy Isoflavone-Treated Eels

**Figure 1:**
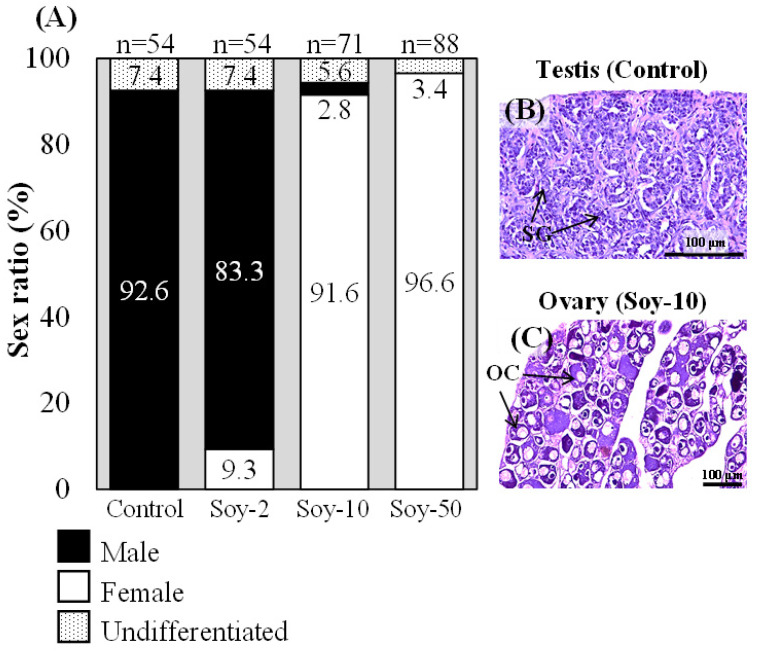
Feminization effects of soy isoflavones in Japanese eels. (**A**) Sex ratios in Japanese eels treated with 2, 10, or 50 g/kg diet of soy isoflavones (Soy). Control, normal diet with no soy isoflavone treatment. (**B**,**C**) Histological sections of gonads after hematoxylin and eosin staining: control eel testis (**B**) and isoflavone-treated eel ovary (**C**). SG, spermatogonium; OC, oocyte.

**Figure 2:**
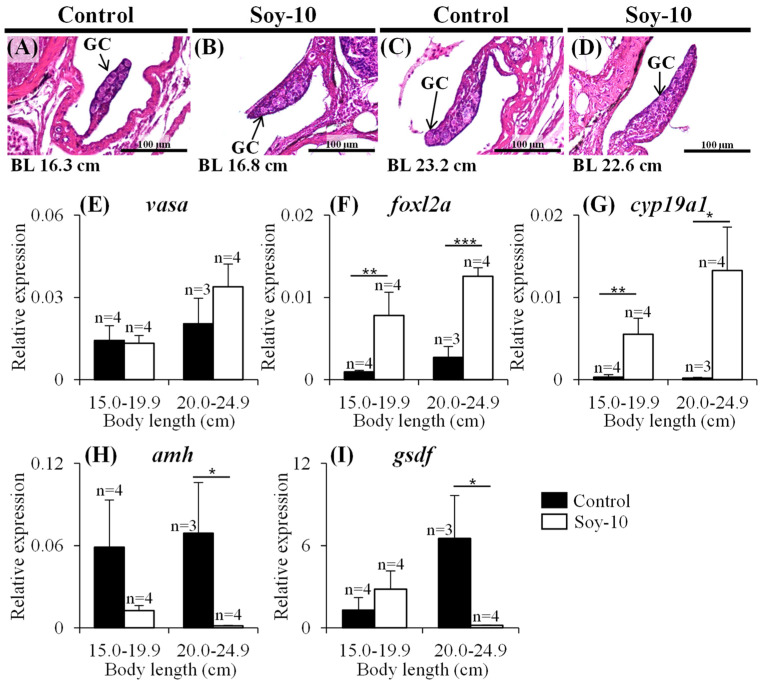
Histological analysis of germ cells and quantitative real-time PCR (qPCR) analysis of sex-specific genes in the gonads of Japanese eels raised to 45 days. (**A**–**D**) Histological sections of undifferentiated gonads after hematoxylin and eosin staining in body-length groups medium (**A**,**B**) and large (**C**,**D**). (**E**–**I**) Expression of sex-specific genes in the gonads of eels by qPCR analysis. Relative expression levels of the target genes were normalized against the expression of elongation factor 1 alpha (*ef1α*): *vasa* (**E**), *foxl2a* (**F**), *cyp19a1* (**G**), *amh* (**H**), and *gsdf* (**I**). * *p* < 0.05; ** *p* < 0.01; *** *p* < 0.001; GC, germ cell.

Japanese eels raised to at least 120 days with body length >30 cm were sacrificed, and their gonadal sex was determined. In the control group, 92.6% (n = 50) of the eels were males, and 7.4% (n = 4) were undifferentiated (Figure 1A). In the soy isoflavone-treated group fed the low dose of soy isoflavone (2 g/kg feed), 9.3% (n = 5) were females, whereas in the groups fed the medium and high doses of soy isoflavone (10 and 50 g/kg feed), 91.6% (n = 65) and 96.6% (n = 85) were females (Figure 1A). Spermatogonia were observed in males in the control groups (Figure 1B), and oocytes were observed in females in the soy isoflavone-treated groups (Figure 1C). To investigate gene expression, the eels were raised to 45 days and then divided into two groups according to body length (medium 15.0–19.9 cm; large 20.0–24.9 cm). In both length groups of the controls and soy isoflavone-treated eels, the germ cells were at the undifferentiated stage (Figure 2A–D). Next, the expression patterns of five sex-specific genes (*vasa*, *foxl2a*, *cyp19a1*, *amh* and *gsdf*) were analyzed by quantitative real-time PCR (qPCR). The germ cell marker *vasa* was expressed in the controls and in the soy isoflavone-treated groups (Figure 2E). Expression of the female-specific genes *foxl2a* and *cyp19a1* were significantly higher in the gonads of the soy isoflavone-treated eels than in those of the controls in both length groups (Figure 2F,G). Conversely, in the large-length groups, expression of the male-specific genes *amh* and *gsdf* was significantly higher in the gonads of the control than in the gonads of the soy isoflavone-treated eels (Figure 2H,I).

### 2.2. Sex Ratio of Genistein- and Daidzein-Treated Japanese Eels

**Figure 3:**
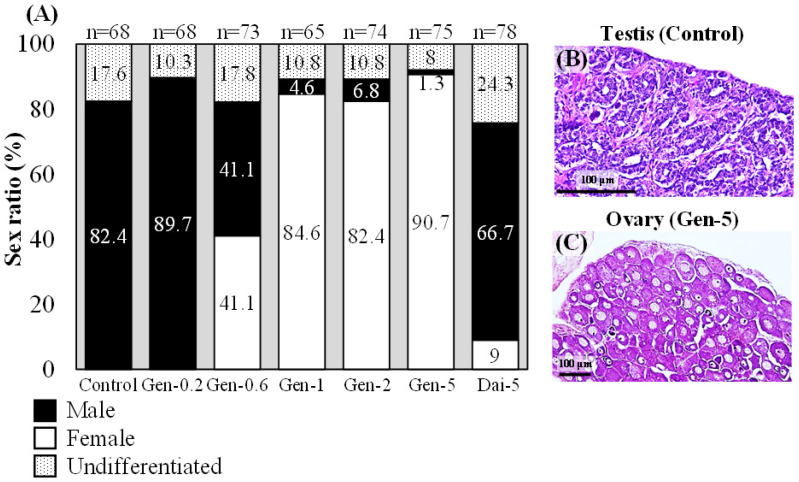
Feminization effects of genistein or daidzein in Japanese eels. (**A**) Sex ratios in eels fed a diet with 0.2, 0.6, 1, 2, or 5 g/kg feed dose of genistein (Gen) or 5 g/kg feed dose of daidzein (Dai). Control, normal diet. (**B**,**C**) Histological sections of gonads after hematoxylin and eosin staining: control eel testis (**B**) and genistein-treated eel ovary (**C**).

A diet with 0.2 g/kg feed dose of genistein resulted in 89.7% males, a diet with 0.6 g/kg feed dose of genistein resulted in 41.1% females (Figure 3A), and diets with 1, 2, and 5 g/kg feed dose of genistein resulted in >80% females (Figure 3A,C). In contrast, a diet with a 5 g/kg feed dose of daidzein resulted in 66.7% of males, and only 9.0% of the eels had ovaries (Figure 3A). The control group consisted of 82.4% males (Figure 3A,B).

### 2.3. RNA Sequencing (RNA-Seq) Analysis of Gonads during Eel Sexual Differentiation

**Figure 4:**
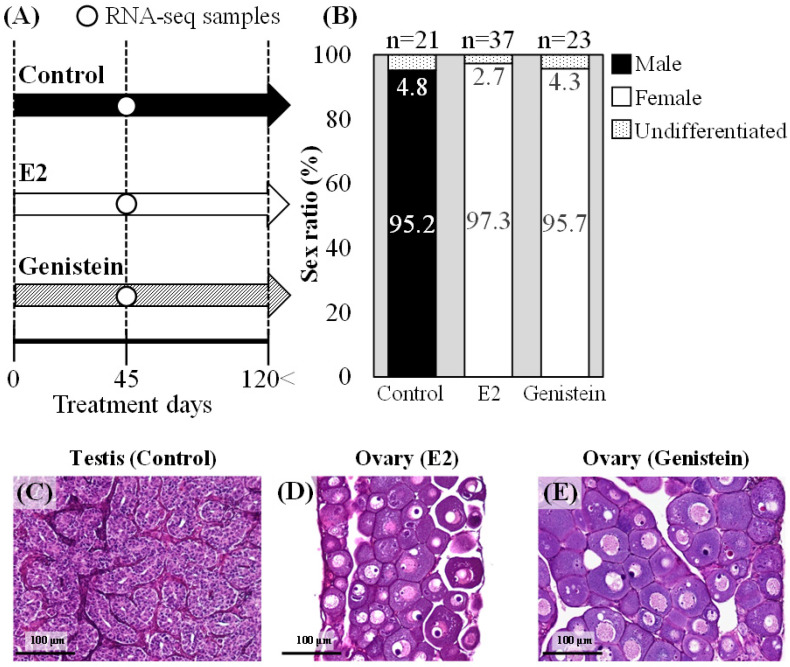
Effects of genistein and estradiol-17β (E2) in Japanese eels. (**A**) Time course of the feminization experiments in the eels fed an E2- or genistein-treated diet or untreated diet (control). (**B**) Sex ratios in the eels fed an E2- or genistein-treated diet or untreated diet (control). (**C**–**E**) Histological sections of gonads after hematoxylin and eosin staining: control testis (**C**), E2-treated eel ovary (**D**), and genistein-treated eel ovary (**E**).

We performed RNA-seq analysis to determine the estrogenic effect of the phytoestrogen genistein on several sex-related genes in Japanese eels compared with the effect of E2. Eels raised to 45 days were collected for gonadal histology (Figure 4A), and all were found to have undifferentiated gonads. Among the eels reared to at least 120 days, 95.2% (n = 20) were males, and 4.8% (n = 1) had undifferentiated gonads in the controls (Figure 4B,C), whereas 97.3% (n = 36) were females, and 2.7% (n = 1) had undifferentiated gonads in the E2-treated eels (Figure 4B,D). Similarly, 95.7% (n = 22) were females, and 4.3% (n = 1) had undifferentiated gonads in the genistein-treated eels (Figure 4B,E).

**Figure 5:**
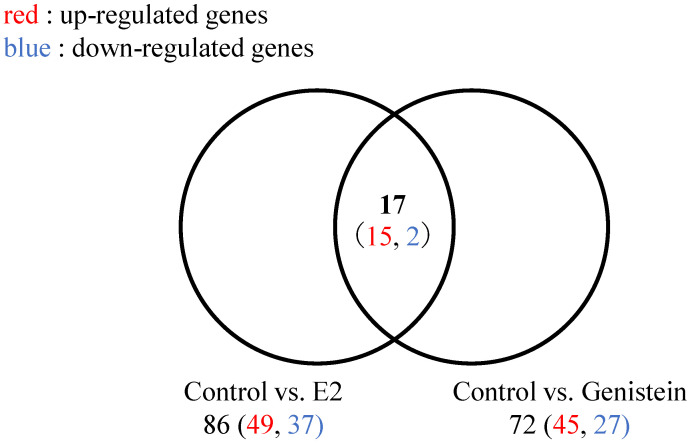
E2- and genistein-induced changes in gene expression. Venn diagram showing the number of differentially expressed genes with expression changes of >10-fold in E2- and genistein-treated eels compared with the genes of control eels.

**Figure 6:**
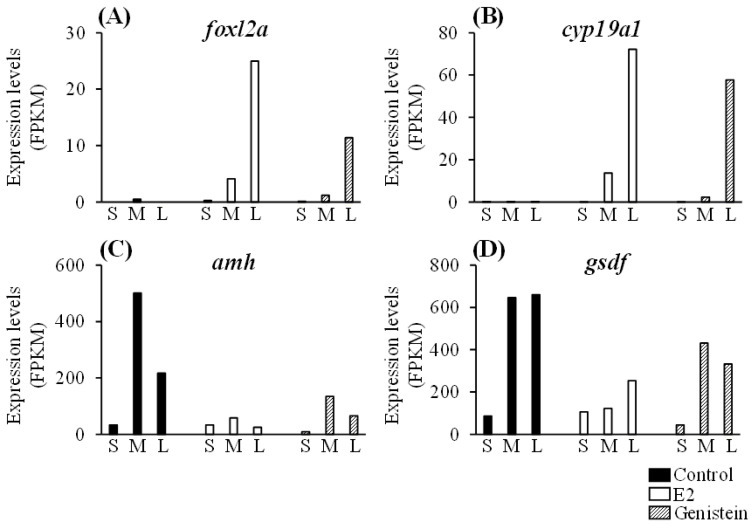
FPKM values of sex-specific genes in the gonads of Japanese eels. (**A**,**B**) Female-specific genes. (**C**,**D**) Male-specific genes. S, small (body length <15.0 cm); M, medium (body length 15.0–19.9 cm); L, large (body length 20.0–24.9 cm).

We focused on genes with fragments per kilobase of exon per million mapped reads (FPKM) values that changed by >10-fold in the E2- and genistein-treated eels compared with the FPKM values in the control group; 86 and 72 such genes were detected in the E2- and genistein-treated eels, respectively (Figure 5, Supplementary Table S1). Among these differentially expressed genes, 15 were up-regulated, and 2 were down-regulated in both the E2- and genistein-treated eels (Figure 5, Table 1). The female-specific genes *foxl2a* and *cyp19a1* had FPKM values that were higher in the gonads of E2- and genistein-treated juveniles than they were in the control eels in the medium and large groups (Figure 6A,B). The male-specific genes *amh* and *gsdf* had FPKM values that were higher in the gonads of the control juveniles than they were in the E2- and genistein-treated eels in the medium and large groups (Figure 6C,D).

Functional and physiological interactions of the differentially expressed genes in E2- and genistein-treated eels were visualized using the STRING database, and the connected networks were detected in each group (Supplementary Figure S1). In both groups, the identified clusters concentrated on the glutamyl prolyl tRNA synthetase (EPRS), which was up-regulated in both E2- and genistein-treated eels, suggesting that EPRS modulates the gene expression.

## 3. Discussion

In this study, sex differentiation of Japanese eels was successfully diverted toward females by adding isoflavones into the feed. Under aquaculture conditions, Japanese eels mostly become males [3,4]. Because females gain higher body weight and obtain better commercial value than males, increasing the percentage of females in a population will be beneficial to the culture of eels. The use of synthetic steroids in fish farming is unlawful and compromises the safety of aquaculture products. Therefore, feminization methods using isoflavones may be useful in eel farming.

Of the isoflavones in soybean and its products, genistein and daidzein are present in the highest concentrations and are the most biologically active of the soy isoflavones [28]. In previous studies, genistein was shown to bind to mammalian and fish estrogen receptors and to have both agonistic and antagonistic effects [21,24]. The complex mechanisms of genistein activity and the physiological responses elicited in in vivo models can result in a mixture of estrogenic and antiestrogenic effects [29,30]. These effects vary greatly among species [31] and depend on the exposure dose, the time of exposure, and other yet unknown factors, possibly including differential activation of estrogen receptor sub-types and enzymes. In the present study, genistein showed a concentration-dependent feminization effect in Japanese eels. In European eels fed genistein at 2 mg/kg for 100 days, 55% were females, but at 20 mg/kg, only 15% were females [27]. Our results are in general agreement with those of other studies, confirming that isoflavones produce estrogenic responses in fish. However, these results also demonstrate the complexity of characterizing an “estrogenic” response because, although genistein produces similar changes in the sexual differentiation of eels, the sensitivity of the response is different for different species. Clearly, the “estrogenic” response requires further study.

We selected the female-specific genes, *foxl2a* and *cyp19a1*, for evaluation. The *foxl2a* gene is a widely known female sexual dimorphism marker that is expressed during ovarian development [32]. *foxl2* is also a positive upstream activator of *cyp19*, which is an androgen-to-estrogen formation activator [33]. Up-regulation of *foxl2* and *cyp19* has been reported in zebrafish, fathead minnow (*Pimephales promelas*), and medaka exposed to various estrogens and xenoestrogens and in fish reproductive endocrine changes [34,35,36,37,38]. It was reported previously that the expression of these genes was increased by E2 in Japanese eels [14]. In the present study, the increased expression of *foxl2a* and *cyp19a1* in genistein-treated eels, similar to E2, suggests that genistein has an estrogenic effect in eels.

In contrast, the expression of the male-specific genes was significantly suppressed in the genistein-treated eels. The *amh* gene has been shown to regulate germ cell proliferation and/or differentiation in male gonad development in different fish species [16,39,40], including Japanese eel [41]. The *gsdf* gene was shown to play a vital role in the development of both testis and ovary in medaka [42,43], and the *gsdf* mRNA expression pattern was found to be related to testes development in gonochoristic fish and to sex change in protogynous fish [44]. Additionally, some studies have reported that the expression of male-specific genes was suppressed by exposure to estrogen-like substances. For example, *gsdf* expression was suppressed by exposure to estradiol benzoate in medaka [38]. In a previous study, we showed that *amh* and *gsdf* expression was suppressed in the gonads of E2-treated Japanese eels compared with their expression in untreated eels [14]. Therefore, although *amh* and *gsdf* expression may be directly regulated by E2 and genistein, no report has been found yet. Future studies will focus on the transcriptional regulatory mechanisms of these genes by E2 and genistein.

To compare the sex-specific genes in E2- and genistein-treated eels during sexual differentiation, we performed a comprehensive RNA-seq expression analysis. None of the 17 detected differentially expressed genes (15 up-regulated, 2 down-regulated) associated with the E2- and genistein treatments were related to sexual differentiation. STRING analysis indicated that the identified clusters concentrated on the EPRS, which was up-regulated in both E2- and genistein-treated eels. EPRS is identified as a tumor immunogen in human breast and gastrointestinal cancers [45]. Recently, it has been reported that EPRS is a critical regulator of cell proliferation and estrogen signaling in ER+ breast cancer [46]. Therefore, EPRS may be involved in the induction of feminization by regulating estrogen signaling in eels. On the other hand, liver X receptor (LXR) was isolated as one of the genes down-regulated in both E2- and genistein-treated eels. LXR is involved in cholesterol homeostasis and glucocorticoid synthesis in mice [47], and cortisol-mediated lipid metabolism pathways have been found to be involved in fish masculinization [48,49]. In a preliminary experiment, we found that treatment of genetic female (XX) medaka with an LXR agonist resulted in 25% males [50], suggesting that LXR-mediated lipid metabolism systems may be involved in the masculinization of cultured eels.

In conclusion, the isoflavone genistein efficiently induced the feminization of Japanese eels. Moreover, the up-regulation of female-specific genes and down-regulation of male-specific genes detected in the gonads suggests that genistein may alter the molecular pathways responsible for eel sex differentiation. Together, our results suggest that genistein has an estrogenic effect on eels similar to E2.

## 4. Materials and Methods

### 4.1. Animals

Elvers of Japanese eels (*Anguilla japonica*) were purchased from the Isshiki Unagi Cooperative Association of Fisheries and transferred to the aquaculture station of the Aichi Fisheries Research Institute. The elvers were acclimated to a freshwater pond environment (indoor 0.8-ton fiberglass tank) with natural lighting, continuous aeration, and warm conditions (28–30 °C) until used.

### 4.2. Experimental Treatments

For the experiment to determine the feminization effects of soy isoflavones and the expression patterns of sex-specific genes, 100 elvers (body length = 7.15 ± 0.99 cm; body weight = 0.33 ± 0.17 g) were placed in each of four replicate 200-L tanks and fed a commercial eel diet containing soy isoflavones (2, 10, 50 g/kg feed) or a control diet with no additives. The soy isoflavones used in this study were genistein (10%) and daidzein (10%). The eels were fed the diet 6 days a week. To determine the expression of the sex-specific genes in the gonads by qPCR, we used control eels and eels fed a diet containing soy isoflavones (10 g/kg feed) for 45 days. The eels were collected and divided into two body-length groups (medium, 15–19.9 cm; large, 20–24.9 cm) for gonadal histology and gene expression analysis. The remaining eels from the four groups were collected after 120 days when most were at least 30 cm. Phenotypic sex was determined by histological observation.

For the experiment to determine the concentration-dependent feminization effects of genistein, 90 elvers (body length = 7.23 ± 0.69 cm; body weight = 0.33 ± 0.12 g) were placed in each of seven replicate 200-L tanks and fed a commercial eel diet containing genistein (Tokyo Chemical Industry Co., Ltd., Tokyo, Japan) (0.2, 0.6, 1, 2, 5 g/kg feed), daidzein (Tokyo Chemical Industry Co., Ltd., Tokyo, Japan) (5 g/kg feed), or a control diet with no additives. The eels were fed the diet 6 days a week. The eels were collected from all the groups after 120 days when most were at least 30 cm. Phenotypic sex was determined by histological observation.

For the experiment to investigate the feminization mechanisms, 100 elvers (body length = 7.57 ± 0.54 cm; body weight = 0.37 ± 0.09 g) were placed in each of three replicate 640-ton fiberglass tanks and fed with a commercial eel diet containing E2 (10 mg/kg feed; Sigma-Aldrich Corp., St. Louis, MO, USA), genistein (1 g/kg feed), or the control diet with no additives. The eels were fed the diet 6 days a week. For comprehensive expression analysis of gonads by RNA-seq, the eels were treated with or without E2 or genistein for 45 days. The eels were collected and divided into three body-length groups (small, <15.0 cm; medium, 15–19.9 cm; large, 20–24.9 cm) for gonadal gene expression analysis. The remaining eels from all the groups were collected after 120 days when most were at least 30 cm.

### 4.3. Quantitative Real-Time PCR (qPCR)

The qPCRs were performed as described previously [14]. Total RNA was extracted from the gonadal tissues of the eels by homogenization in ISOGEN (Nippon Gene, Tokyo, Japan). Reverse transcription was performed at 37 °C for 30 min, using PrimeScript RT Master Mix (TaKaRa, Shiga, Japan). The qPCRs were performed on a Light Cycler 480 system (Roche, Mannheim, Germany) using SYBR Green I Master Mix (Roche). The PCR conditions were 95 °C for 5 min, then 45 cycles of 95 °C for 5 min, 59 °C for 10 s, and 72 °C for 10 s. A melting-curve analysis was conducted after thermo-cycling to evaluate the specificity of the amplification and to verify the absence of primer dimers. Relative gene expression levels were calculated using the ΔΔCT method [51]. The mRNA levels of sex-specific genes (*vasa*, *foxl2a*, *cyp19a1*, *amh* and *gsdf*) were measured using primers as described previously [14]. The copy number values of each gene were normalized against elongation factor 1 alpha (*ef1α*).

### 4.4. Next-Generation Sequencing and RNA-Seq Analysis

Total RNA was extracted from the gonads of the eels as described in Section 4.3. RNA integrity was evaluated using an Agilent 2100 Bioanalyzer system (Agilent Technologies, Santa Clara, CA, USA). An mRNA sequencing library was constructed using a TruSeq RNA Library Prep kit v2 (Illumina, San Diego, CA, USA). Paired-end (2 × 100 bp) sequencing was performed using the Illumina NovaSeq 6000 Sequencing System (Illumina) at the Beijing Genomics Institute. Raw paired-end reads were filtered using the FASTX-Toolkit (http://hannonlab.cshl.edu/fastx_toolkit/index.html). The clean reads were mapped to the Japanese eel genome assembly data (GenBank: BEWY00000000.1) using HISAT2 (v2.1.0), and mapped transcripts were quantified considering multiple splice variants using StringTie (v2.1.7). A count table of FPKM values was created using Cuffdiff (v2.1.1). Functional annotation by Gene Ontology (GO) terms was analyzed using Blast2GO annotation by OmicsBox (v2.2.4). Functional and physiological interactions of the genes were visualized using STRING version 11.5 (https://string-db.org/; accessed on 17 December 2022).

### 4.5. Histological Analysis

The samples of eel body segments or gonadal tissues were fixed in Bouin’s solution, left overnight at 4 °C, then dehydrated, embedded in paraffin, and serially sectioned at 5 µm. The sections were stained with hematoxylin and eosin. Gonadal status was determined by examining the stained tissue sections under a light microscope.

### 4.6. Statistical Analysis

The gene expression analysis results were tested using Levene’s test for homogeneity of variance. Data were compared using Student’s *t*-test or analyzed by one-way ANOVA followed by Tukey’s multiple comparison test, using SPSS Statistics (IBM Corp., Armonk, NY, USA).

## Figures and Tables

**Table 1:** Differentially expressed genes with FPKM values that changed by >10-fold in both E2- and genistein-treated eels compared with the genes of control eels.

	No.	Gene Name	GO Term
**up-regulated genes**	1	QNR-71	membrane
	2	class E basic helix-loop-helix 41	regulation of DNA-templated transcription, cell differentiation, anatomical structure development, DNA binding, transcription regulator activity
	3	phosphatase 1 regulatory subunit 26	negative regulation of phosphatase activity, protein phosphatase inhibitor activity
	4	TBC1 domain family member 9B	calcium ion binding
	5	ATP-dependent DNA helicase Q1	DNA replication, DNA repair, DNA recombination, hydrolase activity, catalytic activity, acting on DNA, ATP-dependent activity, nucleus
	6	Y-box-binding 2	nucleic acid binding
	7	complex I intermediate-associated mitochondrial	generation of precursor metabolites and energy, mitochondrion organization, protein-containing complex assembly, mitochondrion
	8	centromere T	Not Applicable
	9	histidine N-acetyltransferase-like	acetyltransferase activity
	10	transcription intermediary factor 1-alpha-like	Not Applicable
	11	glutamyl prolyl tRNA synthetase	tRNA metabolic process, amino acid metabolic process, ligase activity, catalytic activity, acting on RNA
	12	GTPase IMAP family member 8-like	nucleotide binding, GTP binding
	13	calmodulin-regulated spectrin-associated 2a	cell differentiation, anatomical structure development, cytoskeletal protein binding, cytoskeleton
	14	RNA-binding 14b	RNA binding
	15	L-aminoadipate-semialdehyde dehydrogenase-phosphopantetheinyl transferase	amino acid metabolic process, protein modification process, transferase activity, cytosol
**down-regulated genes**	16	syntaxin-5-like	intracellular protein transport, vesicle-mediated transport, membrane organization, molecular adaptor activity, Golgi apparatus
	17	oxysterols receptor LXR-alpha-like	regulation of DNA-templated transcription, lipid metabolic process, signaling, DNA binding, molecular transducer activity, transcription regulator activity, nucleus

## Data Availability

All data are available within this article.

## Supplementary Materials

The following supporting information can be downloaded at: https://www.mdpi.com/article/10.3390/ijms24010396/s1.
