# Supplementary material for: Soy Isoflavones Induce Feminization of Japanese Eel (Anguilla japonica)
Source: Int J Mol Sci. 2022 Dec 26;24(1):396. doi: 10.3390/ijms24010396 (PMC9820629; doi:10.3390/ijms24010396)
Supplement: Supplementary file 1 [file ijms-24-00396-s001.zip › ijms-2033778-supplementary.pdf]

Soy isoflavones induce feminization of Japanese eel (*Anguilla japonica*)

Supplementary Materials

Hiroyuki Inaba[1, 2, 3], Yuzo Iwata[1, 4], Takashi Suzuki[1, 5], Moemi Horiuchi[6], Ryohei Surugaya[6], Shigeho Ijiri[6], Ai Uchiyama[7], Ryoko Takano[7], Seiji Hara[2, 8], Takashi Yazawa[9] & Takeshi Kitano[2]*

Supplementary Table S1. Differentially expressed genes with FPKM values that changed by >10-fold in E2- and genistein-treated eels compared with the genes of control eels.

	No.	Tracking_id	Gene name	STRING protein	GO Term
	1	MSTRG.10606.3	transcription elongation regulator 1-like isoform X4	LOC797473	P:DNA-templated transcription; F:transcription coregulator activity; F:RNA polymerase binding
	2	MSTRG.10827.2	RNA-binding 14b isoform X3	rbm14b	F:RNA binding
	3	MSTRG.10866.2	hyaluronan mediated motility receptor-like	hmmr	C:cytoskeleton
	4	MSTRG.10894.2	TBC1 domain family member 9B isoform X2	tbc1d9	F:calcium ion binding
	5	MSTRG.11072.2	apoptotic chromatin condensation inducer in the nucleus-like		F:protein heterodimerization activity
	6	MSTRG.11947.1	Y-box-binding 2 isoform X2	ybx1	F:nucleic acid binding
	7	MSTRG.11971.1	histidine N-acetyltransferase-like	nat16l	F:acetyltransferase activity
	8	MSTRG.12631.4	RNA-binding 47-like isoform X2	RBM47	F:RNA binding; C:nucleus
	9	MSTRG.13158.2	ATP-dependent DNA helicase Q1 isoform X2	recql	P:DNA replication; P:DNA repair; P:DNA recombination; F:hydrolase activity; F:catalytic activity, acting on DNA; F:ATP-dependent activity; C:nucleus
	10	MSTRG.13442.2	hypothetical protein	optn	C:cytoplasmic vesicle
	11	MSTRG.13927.2	39S ribosomal protein L2, mitochondrial	MRPL2	P:mitochondrial gene expression; F:RNA binding; F:structural molecule activity; C:mitochondrion; C:ribosome
	12	MSTRG.14896.2	cGMP-dependent 3',5'-cyclic phosphodiesterase isoform X4	pde2a	
	13	MSTRG.14932.2	L-aminoadipate-semialdehyde dehydrogenase-phosphopantetheinyl transferase isoform X3	aasdhppt	P:amino acid metabolic process; P:protein modification process; F:transferase activity; C:cytosol
	14	MSTRG.14942.2	myosin VIIAa isoform X3	myo7aa	P:vesicle-mediated transport; P:cell differentiation; P:anatomical structure development; P:nervous system process; F:cytoskeletal motor activity; F:cytoskeletal protein binding; C:cytoskeleton
	15	MSTRG.15005.2	probable G- coupled receptor 171	GPR171	P:signaling; F:molecular transducer activity
	16	MSTRG.15326.2	5'-3' exonuclease PLD3	pld3	P:regulation of DNA-templated transcription; P:anatomical structure development; F:DNA binding; F:catalytic activity; F:transcription regulator activity; C:nucleus
	17	MSTRG.16127.3	cleavage stimulation factor subunit 1	cstf1	P:mRNA metabolic process; F:RNA binding; F:translation regulator activity; C:nucleus
	18	MSTRG.16702.2	---NA---		
	19	MSTRG.17137.2	---NA---		
	20	MSTRG.18405.2	nuclear factor related to kappa-B-binding protein isoform X2	NFRKB	C:nuclear chromosome
	21	MSTRG.19232.2	transcriptional regulator ATRX-like	atrx	C:organelle
	22	MSTRG.20405.1	queuine tRNA-ribosyltransferase catalytic subunit 1 isoform X1	qtrt1	P:tRNA metabolic process; F:transferase activity; F:catalytic activity, acting on RNA; C:mitochondrion
	23	MSTRG.20758.3	class E basic helix-loop-helix 41 isoform X1	bhlhe41	P:regulation of DNA-templated transcription; P:cell differentiation; P:anatomical structure development; F:DNA binding; F:transcription regulator activity
	24	MSTRG.21449.1	parvalbumin alpha	pvalb3	F:calcium ion binding
**E2 up-regulated genes**	25	MSTRG.21913.2	epsin-3-like isoform X2	epn3b	F:lipid binding
	26	MSTRG.2248.2	centromere T isoform X2	CENPT	
	27	MSTRG.22606.3	LOW QUALITY PROTEIN: afadin-like	afdna	P:cell adhesion; P:signaling; P:cell differentiation; P:anatomical structure development
	28	MSTRG.23317.3	protein SMG7 isoform X8	smg7	
	29	MSTRG.23423.2	zinc finger 644	znf644b	P:regulation of DNA-templated transcription; F:DNA binding; F:transcription regulator activity; C:nucleus
	30	MSTRG.23864.2	spindlin-W-like	LOC798602	P:reproductive process
	31	MSTRG.23972.6	---NA---		
	32	MSTRG.26525.1	phosphatase 1 regulatory subunit 26 isoform X1	ppp1r26	P:negative regulation of phosphatase activity; F:protein phosphatase inhibitor activity
	33	MSTRG.26603.2	peroxisomal NADH pyrophosphatase NUDT12 isoform X1	nudt12	F:hydrolase activity; C:peroxisome
	34	MSTRG.26951.4	serine/threonine-protein kinase MARK2-like isoform X9	mark2b	
	35	MSTRG.27670.2	adenosine 3 -phospho 5 -phosphosulfate transporter 1 isoform X2	slc35b2	P:cell differentiation; P:extracellular matrix organization; P:anatomical structure development; P:transmembrane transport; P:carbohydrate derivative metabolic process; F:transporter activity
	36	MSTRG.29080.2	GTPase IMAP family member 8-like	LOC560863	F:nucleotide binding; F:GTP binding
	37	MSTRG.32378.1	QNR-71 isoform X1	gpnmb	C:membrane; C:membrane
	38	MSTRG.33375.2	uncharacterized protein rusc1 isoform X2	LOC558727	
	39	MSTRG.33384.3	DNA-directed RNA polymerase III subunit RPC3 isoform X1	polr3c	P:DNA-templated transcription; F:DNA binding; C:nucleus
	40	MSTRG.3469.2	LOW QUALITY PROTEIN: erbin	erbb2ip	P:regulation of DNA-templated transcription; P:signaling
	41	MSTRG.4812.6	tRNA selenocysteine 1-associated 1-like	C2H6orf52	F:nucleic acid binding; C:intracellular anatomical structure
	42	MSTRG.5386.1	transcription intermediary factor 1-alpha-like isoform X2	trim33l	
	43	MSTRG.5780.3	ceruloplasmin isoform X1	cp	F:oxidoreductase activity; C:extracellular region
	44	MSTRG.6174.1	coagulation factor XIII A chain-like isoform X1	f13a1b	
	45	MSTRG.6300.1	calmodulin-regulated spectrin-associated 2a isoform X3	camsap2	P:cell differentiation; P:anatomical structure development; F:cytoskeletal protein binding; C:cytoskeleton
	46	MSTRG.7318.2	F-actin-monooxygenase mical1 isoform X4	mical1	P:anatomical structure development; F:cytoskeletal protein binding; F:oxidoreductase activity
	47	MSTRG.25329.2	glutamyl prolyl tRNA synthetase	eprs	P:tRNA metabolic process; P:amino acid metabolic process; F:ligase activity; F:catalytic activity, acting on RNA
	48	MSTRG.8730.2	complex I intermediate-associated mitochondrial	ndufaf1	P:generation of precursor metabolites and energy; P:mitochondrion organization; P:protein-containing complex assembly; C:mitochondrion
	49	MSTRG.9599.2	E3 ubiquitin- ligase HACE1 isoform X1	hace1	P:protein modification process; F:transferase activity; F:ligase activity; F:catalytic activity, acting on a protein
	1	MSTRG.11227.2	zinc finger CCHC domain-containing 7 isoform X2	zcchc7	F:nucleic acid binding; F:zinc ion binding
	2	MSTRG.12780.2	palmitoyltransferase ZDHHC5a	zdhhc5a	P:protein modification process; F:transferase activity; F:catalytic activity, acting on a protein; C:plasma membrane
	3	MSTRG.13765.3	inactive carboxypeptidase-like protein X2	cpxm1a	P:protein maturation; F:hydrolase activity; F:catalytic activity, acting on a protein; C:extracellular space
	4	MSTRG.13944.2	BRD4-interacting chromatin-remodeling complex-associated -like	gltscr1l	P:positive regulation of DNA-templated transcription; C:SWI/SNF complex
	5	MSTRG.15553.2	protein-serine O-palmitoleoyltransferase porcupine-like isoform X2	porcn	P:lipid metabolic process; P:signaling; P:protein modification process; F:transferase activity; C:endoplasmic reticulum
	6	MSTRG.15920.2	microtubule-associated RP EB family member 1-like	mapre1b	F:cytoskeletal protein binding; C:cytoskeleton
	7	MSTRG.16100.6	membrane-associated guanylate WW and PDZ domain-containing 1b isoform X8	magi1b	F:transferase activity
	8	MSTRG.16846.2	uveal autoantigen with coiled-coil domains and ankyrin repeats -like isoform X1	sb:cb472	P:programmed cell death; P:signaling; C:cytoskeleton
	9	MSTRG.17606.2	dnaJ homolog subfamily B member 2 isoform X5	dnajb2	
	10	MSTRG.17679.2	very-long-chain (3R)-3-hydroxyacyl-CoA dehydratase 2-like isoform X1	ptplb	P:lipid metabolic process; F:lyase activity; C:endoplasmic reticulum
	11	MSTRG.18773.2	rho GTPase-activating 6-like	ARHGAP36	P:signal transduction
	12	MSTRG.19188.2	syntaxin-5-like	stx5al	P:intracellular protein transport; P:vesicle-mediated transport; P:membrane organization; F:molecular adaptor activity; C:Golgi apparatus
	13	MSTRG.19232.3	transcriptional regulator ATRX-like	atrx	C:organelle
	14	MSTRG.20961.2	---NA---		
	15	MSTRG.21244.2	serine threonine- kinase MARK1 isoform X2	zgc:153725	P:cytoskeleton organization; P:protein modification process; F:transferase activity; F:catalytic activity, acting on a protein
	16	MSTRG.2155.2	---NA---		
	17	MSTRG.22116.2	retinoic acid-induced 1-like	LOC560882	F:metal ion binding
	18	MSTRG.23027.2	DIS3-like exonuclease 2	LOC566106	P:mRNA metabolic process; F:RNA binding; F:hydrolase activity; F:catalytic activity, acting on RNA; C:organelle
**E2 down-regulated genes**	19	MSTRG.23134.3	activated CDC42 kinase 1-like isoform X5	LOC559119	P:signaling; P:protein modification process; F:transferase activity; F:catalytic activity, acting on a protein; C:nucleus; C:plasma membrane
	20	MSTRG.2324.2	STRA6-like isoform X1	zgc:162946	C:membrane; C:membrane
	21	MSTRG.24294.2	beta-1,3-galactosyl-O-glycosyl-glyco beta-1,6-N-acetylglucosaminyltransferase-like	gcnt3	F:transferase activity; F:catalytic activity, acting on a protein; C:Golgi apparatus
	22	MSTRG.24889.5	cordon-bleu protein-like 1 isoform X1	cobll1b	P:signal transduction; F:actin monomer binding
	23	MSTRG.2524.2	DNA damage-binding 2 isoform X3	ddb2	P:DNA repair; F:damaged DNA binding; C:nucleus; C:Cul4-RING E3 ubiquitin ligase complex
	24	MSTRG.2562.2	oxysterols receptor LXR-alpha-like	nr1h3	P:regulation of DNA-templated transcription; P:lipid metabolic process; P:signaling; F:DNA binding; F:molecular transducer activity; F:transcription regulator activity; C:nucleus
	25	MSTRG.27679.2	BRO1 domain-containing protein BROX	brox	C:membrane
	26	MSTRG.29891.3	NLR family CARD domain-containing 3-like isoform X2	si:ch211-196h16.4	
	27	MSTRG.31319.2	---NA---		
	28	MSTRG.32854.2	protein NDRG2	ndrg2	P:signaling; P:cell differentiation; P:anatomical structure development
	29	MSTRG.33022.3	two pore calcium channel 1-like isoform X6	tpcn1	P:transmembrane transport; F:transporter activity; C:lysosome
	30	MSTRG.3321.2	electrogenic sodium bicarbonate cotransporter 1 isoform X3	slc4a4a	P:transmembrane transport; F:transporter activity
	31	MSTRG.487.1	chromobox protein homolog 8-like	cbx7a	P:regulation of DNA-templated transcription; P:protein modification process; F:transferase activity; F:catalytic activity, acting on a protein; C:nucleus
	32	MSTRG.5071.2	ubiquitin-conjugating enzyme E2Q-like protein 1	UBE2QL1	P:protein modification process; F:transferase activity; F:catalytic activity, acting on a protein; C:nucleus
	33	MSTRG.5214.3	antizyme inhibitor 1-like isoform X1	azin1b	P:polyamine biosynthetic process; P:regulation of catalytic activity; F:catalytic activity; F:ornithine decarboxylase activator activity
	34	MSTRG.8042.3	brefeldin A-inhibited guanine nucleotide-exchange 2	arfgef2	
	35	MSTRG.8169.1	chymotrypsin-like elastase family member 2A	ela2l	F:hydrolase activity; F:catalytic activity, acting on a protein; C:extracellular space
	36	MSTRG.9323.2	vacuolar 8-like isoform X1		F:molecular adaptor activity; C:vacuole
	37	MSTRG.9931.4	interferon-stimulated 20 kDa exonuclease-like 2	isg20l2	P:nucleic acid phosphodiester bond hydrolysis; P:RNA phosphodiester bond hydrolysis, exonucleolytic; F:3'-5'-exoribonuclease activity; F:nucleic acid binding; F:exonuclease activity
	1	MSTRG.537.1	amine sulfotransferase-like	sult3st3	F:transferase activity
	2	MSTRG.11770.3	arf-GAP with Rho-GAP ANK repeat and PH domain-containing 3 isoform X1	arap3	P:signaling; P:anatomical structure development; F:lipid binding; F:molecular function regulator activity
	3	MSTRG.20743.2	ATP-binding cassette sub-family C member 9 isoform X1	abcc9	P:transmembrane transport; F:transporter activity; F:molecular transducer activity; F:ATP-dependent activity
	4	MSTRG.13158.2	ATP-dependent DNA helicase Q1 isoform X2	recql	P:DNA replication; P:DNA repair; P:DNA recombination; F:hydrolase activity; F:catalytic activity, acting on DNA; F:ATP-dependent activity; C:nucleus
	5	MSTRG.25329.2	glutamyl prolyl tRNA synthetase	eprs	P:tRNA metabolic process; P:amino acid metabolic process; F:ligase activity; F:catalytic activity, acting on RNA
	6	MSTRG.6300.1	calmodulin-regulated spectrin-associated 2a isoform X3	camsap2	P:cell differentiation; P:anatomical structure development; F:cytoskeletal protein binding; C:cytoskeleton
	7	MSTRG.2248.2	centromere T isoform X2	CENPT	
	8	MSTRG.20758.3	class E basic helix-loop-helix 41 isoform X1	bhlhe41	P:regulation of DNA-templated transcription; P:cell differentiation; P:anatomical structure development; F:DNA binding; F:transcription regulator activity
	9	MSTRG.8730.2	complex I intermediate-associated mitochondrial	ndufaf1	P:generation of precursor metabolites and energy; P:mitochondrion organization; P:protein-containing complex assembly; C:mitochondrion
	10	MSTRG.18806.1	coronin-6	coro6	P:cytoskeleton organization; P:protein modification process; P:protein-containing complex assembly; F:cytoskeletal protein binding; F:hydrolase activity; F:catalytic activity, acting on a protein
	11	MSTRG.22690.2	dermatan-sulfate epimerase isoform X1	dse	F:isomerase activity
	12	MSTRG.11283.2	ephrin type-A receptor 3-like isoform X3	epha3l	P:signaling; P:protein modification process; F:transferase activity; F:molecular transducer activity; F:catalytic activity, acting on a protein; C:plasma membrane
	13	MSTRG.24421.2	glutamine--fructose-6-phosphate aminotransferase [isomerizing] 2-like isoform X1	gfpt2	P:amino acid metabolic process; P:nucleobase-containing small molecule metabolic process; P:carbohydrate derivative metabolic process; F:transferase activity
	14	MSTRG.29080.2	GTPase IMAP family member 8-like	LOC560863	F:nucleotide binding; F:GTP binding
	15	MSTRG.22576.2	headcase homolog isoform X1	heca	
	16	MSTRG.11971.1	histidine N-acetyltransferase-like	nat16l	F:acetyltransferase activity
	17	MSTRG.25462.2	hormone-sensitive lipase isoform X3	lipeb	
	18	MSTRG.22947.1	isthmin-2	ism2a	
	19	MSTRG.14932.2	L-aminoadipate-semialdehyde dehydrogenase-phosphopantetheinyl transferase	aasdhppt	P:amino acid metabolic process; P:protein modification process; F:transferase activity; C:cytosol
	20	MSTRG.12628.2	LIM and calponin homology domains-containing 1-like	limch1b	
	21	MSTRG.19454.2	LOW QUALITY PROTEIN: angiomotin	amot	P:establishment or maintenance of cell polarity; P:anatomical structure development; P:cell motility
	22	MSTRG.25475.3	meprin A subunit beta-like isoform X1	mep1b	F:hydrolase activity; F:catalytic activity, acting on a protein
**Genistein up-regulated genes**	23	MSTRG.3560.2	methyltransferase C9orf114	zgc:101748	P:RNA-mediated gene silencing; F:RNA binding; F:transferase activity; C:chromosome; C:microtubule organizing center
	24	MSTRG.828.5	myocardin isoform X2	MYOCD	
	25	MSTRG.828.1	myocardin isoform X6	MYOCD	
	26	MSTRG.16702.2	---NA---		
	27	MSTRG.12523.4	---NA---		
	28	MSTRG.26706.2	---NA---		
	29	MSTRG.17178.2	nuclear factor of activated T- cytoplasmic 3-like isoform X2	NFATC3	P:regulation of DNA-templated transcription; P:signaling; F:DNA binding; F:transcription regulator activity
	30	MSTRG.26525.1	phosphatase 1 regulatory subunit 26 isoform X1	ppp1r26	P:negative regulation of phosphatase activity; F:protein phosphatase inhibitor activity
	31	MSTRG.15056.1	PREDICTED: uncharacterized protein C11orf57 homolog	zgc:162339	
	32	MSTRG.12596.1	PREDICTED: uncharacterized protein LOC106964325 isoform X42		
	33	MSTRG.14401.1	progestogen receptor type 2	pgr	P:regulation of DNA-templated transcription; F:DNA binding; F:transcription regulator activity; C:nucleus
	34	MSTRG.23905.2	proline-rich 12	LOC557848	
	35	MSTRG.13179.1	protein artemis	dclre1c	C:nucleus
	36	MSTRG.10911.15	protocadherin alpha-3-like isoform X13	pcdh2ac	
	37	MSTRG.32378.1	QNR-71 isoform X1	gpnmb	C:membrane; C:membrane
	38	MSTRG.10827.2	RNA-binding 14b isoform X3	rbm14b	F:RNA binding
	39	MSTRG.10894.2	TBC1 domain family member 9B isoform X2	tbc1d9	F:calcium ion binding
	40	MSTRG.5386.1	transcription intermediary factor 1-alpha-like isoform X2	trim33l	
	41	MSTRG.6234.2	uncharacterized protein si:ch211-161h7.4 isoform X1	si:ch211-161h7.4	
	42	MSTRG.26034.1	uncharacterized protein si:ch73-303b9.1		
	43	MSTRG.11947.1	Y-box-binding 2 isoform X2	ybx1	F:nucleic acid binding
	44	MSTRG.57.5	zinc finger 501-like	znf1035	
	45	MSTRG.19144.2	zona pellucida sperm-binding 3-like	zp3a.2	P:reproductive process; P:cell differentiation; P:anatomical structure development; F:structural molecule activity; C:extracellular region; C:nuclear envelope; C:plasma membrane; C:extracellular matrix
	1	MSTRG.10064.2	60S ribosomal protein L18	rpl18	F:structural molecule activity; C:endoplasmic reticulum; C:ribosome
	2	MSTRG.11554.1	C-X-C chemokine receptor type 4a	cxcr4a	P:immune system process; P:signaling; P:cell differentiation; P:anatomical structure development; P:cell motility; F:molecular transducer activity; C:endosome; C:plasma membrane
	3	MSTRG.11770.2	arf-GAP with Rho-GAP domain, ANK repeat and PH domain-containing protein 3	arap3	P:signaling; P:anatomical structure development; F:lipid binding; F:molecular function regulator activity
	4	MSTRG.16741.2	A-kinase anchor protein 13-like	akap13	P:proteolysis; P:regulation of Rho protein signal transduction; P:regulation of catalytic activity; F:metallocarboxypeptidase activity; F:cAMP-dependent protein kinase activity; F:guanyl-nucleotide exchange factor activity; F:zinc ion binding; F:heme binding; C:cytoplasm; C:cytosol
	5	MSTRG.18209.5	flavin-containing monooxygenase FMO GS-OX-like 4	LOC793236	F:oxidoreductase activity; C:organelle
	6	MSTRG.18792.2	inositol polyphosphate 5-phosphatase K isoform X1	inpp5k	P:lipid metabolic process; F:hydrolase activity
	7	MSTRG.19188.2	syntaxin-5-like	stx5al	P:intracellular protein transport; P:vesicle-mediated transport; P:membrane organization; F:molecular adaptor activity; C:Golgi apparatus
	8	MSTRG.20743.1	ATP-binding cassette sub-family C member 9 isoform X1	abcc9	P:transmembrane transport; F:transporter activity; F:molecular transducer activity; F:ATP-dependent activity
	9	MSTRG.21312.3	ribosome biogenesis protein BMS1 homolog isoform X2	bms1l	P:ribosome biogenesis; C:nucleolus
	10	MSTRG.25256.4	---NA---		
	11	MSTRG.2562.2	oxysterols receptor LXR-alpha-like	nr1h3	P:regulation of DNA-templated transcription; P:lipid metabolic process; P:signaling; F:DNA binding; F:molecular transducer activity; F:transcription regulator activity; C:nucleus
	12	MSTRG.25778.3	N-acetyllactosaminide beta-1,3-N-acetylglucosaminyltransferase 2	b3gnt2b	P:protein glycosylation; F:transferase activity; C:Golgi apparatus
	13	MSTRG.26525.3	protein phosphatase 1 regulatory subunit 26 isoform X2	ppp1r26	P:negative regulation of phosphatase activity; F:protein phosphatase inhibitor activity
**Genistein down-regulated genes**	14	MSTRG.26829.4	protein ABHD18	C14H4orf29	
	15	MSTRG.27512.2	alpha-mannosidase 2C1	LOC569595	P:carbohydrate metabolic process; F:hydrolase activity
	16	MSTRG.3039.1	---NA---		
	17	MSTRG.32651.1	NLR family CARD domain-containing protein 3-like		
	18	MSTRG.3495.2	Werner syndrome ATP-dependent helicase isoform X1	wrn	F:hydrolase activity; F:ATP-dependent activity
	19	MSTRG.4665.2	UPF0489 protein C5orf22 homolog	C2H5orf22	
	20	MSTRG.5577.2	transcription termination factor 1, mitochondrial	MTERF1	P:regulation of DNA-templated transcription; P:mitochondrial gene expression; F:DNA binding; C:mitochondrion
	21	MSTRG.5924.2	cyclic AMP-responsive element-binding protein 3-like protein 3-A isoform X3	creb3l3a	P:regulation of DNA-templated transcription; F:DNA binding; C:organelle
	22	MSTRG.6098.2	sushi, nidogen and EGF-like domain-containing protein 1	sned1	P:cell adhesion; P:cell differentiation; P:anatomical structure development
	23	MSTRG.6668.3	uncharacterized protein LOC115112262 isoform X4		
	24	MSTRG.8602.3	free fatty acid receptor 2-like	si:ch73-90p23.1	P:signaling; F:molecular transducer activity
	25	MSTRG.8682.2	synaptosomal-associated protein 23-like isoform X1	zgc:56072	P:vesicle-mediated transport; P:signaling; P:membrane organization; P:protein-containing complex assembly; F:molecular adaptor activity; C:plasma membrane
	26	MSTRG.9229.2	tubulin epsilon and delta complex protein 1	LOC570574	
	27	MSTRG.9540.2	myelin basic protein	mbpa	

(A)

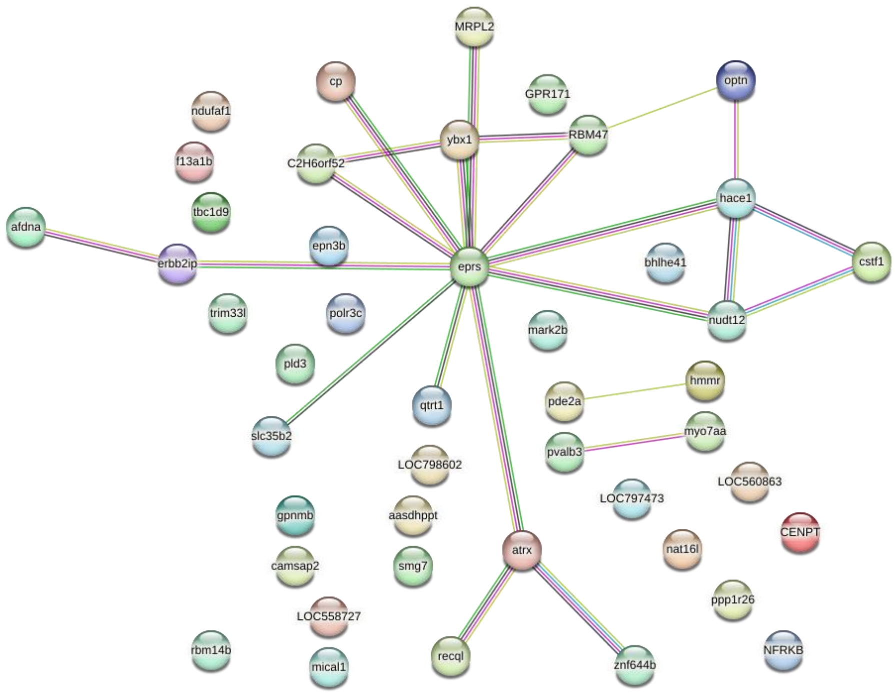

(B)

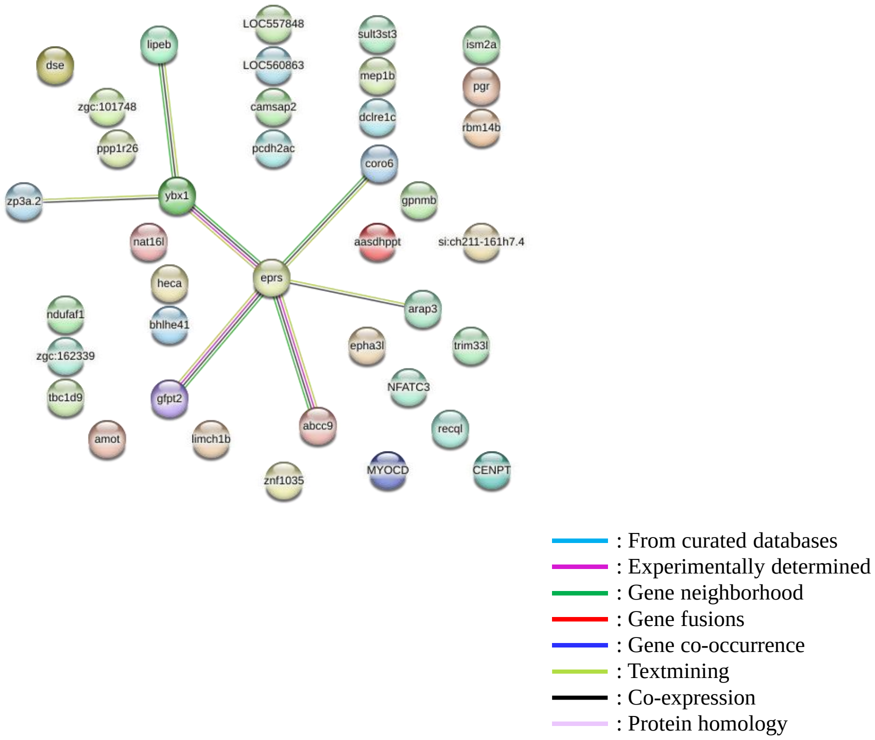

Supplementary Figure S1. Functional and physiological networks visualized by STRING.
(A) Functional and physiological networks within the 49 genes up-regulated by E2
(B) Functional and physiological networks within the 45 genes up-regulated by Genistein
